# rhEGF-Loaded Hydrogel in the Treatment of Chronic Wounds in Patients with Diabetes: Clinical Cases

**DOI:** 10.3390/gels8080523

**Published:** 2022-08-20

**Authors:** Beatriz Guitton Renaud Baptista de Oliveira, Bianca Campos Oliveira, Gabriela Deutsch, Fernanda Soares Pessanha, Rossana Mara da Silva Moreira Thiré, Selma Rodrigues de Castilho

**Affiliations:** 1 Aurora de Afonso Costa School of Nursing, Universidade Federal Fluminense, Niterói 24210-201, Brazil; 2 Faculty of Pharmacy, Universidade Federal Fluminense, Niterói 24210-201, Brazil; 3 Faculty of Nursing, Rio de Janeiro State University, Niterói 24210-201, Brazil; 4 COPPE/Program of Metallurgical and Materials Engineering—PEMM, University of Rio de Janeiro, Rio de Janeiro 21941-599, Brazil

**Keywords:** leg ulcer, diabetic foot, wound healing, epidermal growth factor, nursing

## Abstract

The aim of the study was to evaluate the healing process of chronic wounds treated with carboxymethylcellulose loaded with recombinant human epidermal growth factor in patients with diabetes. The case series consisted of 10 patients treated at the university hospital for 12 weeks. Data were analyzed using SPSS version 22.0. according to the intention to treat the principle, without the loss or exclusion of the participants. The sample consisted of 70% (7/10) males with a mean age of 61.9 years (±9.4); all (100%) had diabetes mellitus and 70% (7/10) had systolic hypertension associated with diabetes mellitus. Sixty percent (6/10) presented lesions of diabetic etiology and 40% (4/10) presented lesions of venous etiology; 70% (7/10) had had lesions for less than 5 years. The mean glycated hemoglobin was 7.8% (±2.7%), while the mean ankle-arm index (AAI) was 0.94 (±0.21). The mean initial area of all wounds was 13.4 cm², and the mean final area was 7.8 cm^2^, with a reduction rate of 28.9% over the 12 weeks of treatment. The reduction rate of diabetic ulcers was higher (33.4%) than that of venous ulcers (22.1%). Regarding the type of tissue, there was an increase in granulation and epithelialization, and a decrease in slough and the amount of exudate that were statistically significant (*p* = 0.021). No participant had severe or local adverse events during the study period. Epidermal growth factor was effective in the treatment of chronic wounds, especially diabetic ulcers, resulting in the reduction of the wound area and the improvement of tissue and exudate quality.

## 1. Introduction

Chronic wounds show delayed wound healing, do not present anatomical and functional skin integrity within 12 weeks, and have high rates of recurrence. This is due to factors such as inadequate angiogenesis and impaired cell perfusion and migration. Examples of chronic wounds include venous, ischemic, diabetic, or pressure ulcers and infected wounds [1,2].

Patients with chronic wounds in Brazil, in addition to the pathologies that are difficult to heal, also suffer from underlying diseases such as venous insufficiency, diabetes mellitus, and systemic arterial hypertension, among other systemic factors that may contribute to the poor healing of wounds such as poor nutritional status, immunosuppression, infection, and socioeconomic factors [3,4].

Several dressings are currently available in the world for the treatment of chronic wounds; these dressings include those containing recombinant human epidermal growth factor (rhEGF). rhEGF is a masterful bioidentical peptide product as it is synthesized using the biotechnological fermentation process of *E. coli* bacteria [5].

Its mechanism of action occurs at the cellular level through interaction with the receptor tyrosine kinase EGF-R, triggering a cascade reaction with signaling that results in a succession of biochemical changes. At the cutaneous level, its effects are manifested by the proliferation of keratinocytes, the stimulation of angiogenesis, and the activation of fibroblastic function with increased levels of endogenous hyaluronic acid, collagen, and elastin [6]. When applied topically, it stimulates gene expression (upregulation), cell proliferation, and differentiation, and can accelerate the healing process and re-epithelialization of chronic wounds, making it clinically relevant for wound healing [7,8].

Despite the promising results of the use of growth factors for skin repair, the choice of appropriate delivery systems has limited the translation of laboratory results to the clinic. An appropriate release system is essential for these biomolecules to remain bioactive for a long time and thus guarantee a satisfactory wound-healing effect. Particularly in the case of rhEGF, this molecule tends to degrade when exposed to proteinase and oxygen in the wound environment. Thus, the rhEGF release system must withstand the application pressure of a dressing to avoid detachment between rhEGF and the wound [9,10].

In recent years, hydrogels have been used to produce wound dressings associated with drug delivery vehicles. These are cross-linked polymers that can be natural or synthetic, can expand in water, and contain a high concentration of water. In general, hydrogels are biodegradable, biocompatible, and non-toxic in situ. They can supply water to the wound site, helping keep the environment moist and supporting quicker wound healing. They can also induce the expression of factors that accelerate the wound healing and tissue regeneration processes [11,12].

In this study, we evaluated the carboxymethylcellulose (CMC) hydrogel as the excipient for rhEGF. CMC-based dressings are known for their flexibility, high exudate adsorption capacity, biocompatibility, and skin-cell-proliferation-inducing ability [13].

Thus, the objective of this study was to evaluate the healing process of chronic wounds treated with a carboximethylcellulose hydrogel loaded with recombinant human epidermal growth factor in patients with diabetes.

## 2. Results

Ten diabetic participants with chronic wounds including six diabetic ulcers and four venous ulcers were included. Each participant was followed for 12 weeks, for a total of 120 nursing consults. All participants received treatment and were evaluated for primary and secondary outcomes, with no loss or exclusion of participants during the follow-up period.

Table 1 shows the main statistics for the clinical and sociodemographic characteristics. The patients in this sample were mostly male (70.0%), with a mean age of 61.9 years (±9.4 years) and presented lesions with diabetic etiology (60.0%) and venous etiology (40.0%) that had been present for less than 5 years (70.0%). Regarding the underlying diseases, all participants had diabetes mellitus (100.0%), and the majority had systolic arterial hypertension associated with diabetes mellitus (70.0%). The patients had a mean BMI of 32.1 kg/m^2^ (±6.3 kg/m^2^), which is considered as Grade 1 Obesity according to the parameters proposed by the Brazilian Association for the Study of Obesity and Metabolic Syndrome (ABESO, for its initials in Portuguese).

The mean glycated hemoglobin level of the patients remained at 7.8% (±2.7%), while the mean ankle-arm index (AAI) was 0.94 (±0.21). The patients showed low variability for age and glycated hemoglobin (coefficients of variation (CV) lower than 0.2), moderate variability for AAI and BMI (0.2 ≤ CV < 0.4), and high variability for lesion duration (CV = 1.17).

**Figure 1:**
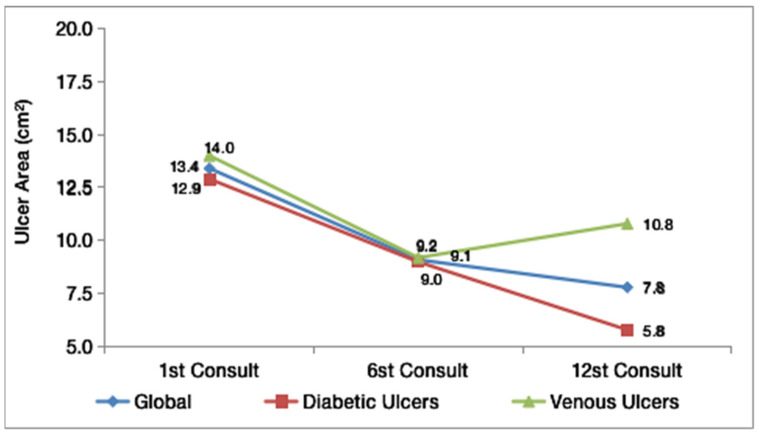
The evolution of the mean area of the lesion in the three evaluations.

Figure 1 shows the progression of the mean wound area (in cm^2^) at weeks 1, 6, and 12 for the 10 patients and for the subgroups stratified according to the etiology of the lesion. The results show reductions in the lesion area for all patients (*n* = 10). These results suggest that there was a reduction between the first visit and the 12th visit, with an overall mean initial area of 13.4 cm^2^ and an overall mean final area of 7.8 cm^2^. Patients with diabetes exhibited the greatest reduction, from 12.9 cm^2^ to 5.8 cm^2^.

Table 2 shows the main statistics for the absolute reduction and relative reduction of the patients’ ulcers. In the overall sample and in the subgroups, the reductions exhibited a high variability (CV greater than 0.4). The *p*-value of the *t*-test of significance for the observed reduction was equal to 0.164, showing that the mean reduction observed in this sample was not statistically significant. In fact, in four cases (40% of the sample), the reduction was less than 1 cm^2^, and the mean was inflated by one patient, who showed a reduction of 38 cm^2^ in the ulcer area. The statistics and graphs suggest that the reduction was greater in the subgroup with diabetic ulcers, but the significance of the difference between the two subgroups could not be confirmed by the tests due to the small size of the subgroups.

Table 3 shows the frequency distribution of the percentages of granulation tissue, epithelialization, and devitalized tissue of the 10 patients in the 12 weeks of treatment.

Initially, the ulcers did not present necrotic tissue, as this was an exclusion criterion to participate in the study, and the presence of this type of tissue was not recorded in any of the ulcers throughout the analyzed period. The granulation tissue was present in all ulcers in all evaluations. Epithelialization was not found in any patient during the first visit and was recorded on the 12th visit for 40% of the patients. Devitalized tissue was found in 70% of the patients at the first visit, and it was found in 40% of the patients at the last evaluation.

The Wilcoxon test was used to perform pairwise comparisons of the initial and final distributions of the percentages of granulation tissue, epithelialization, and devitalized tissue. All results were greater than 5%, indicating that the changes in granulation tissue, epithelialization, and devitalized tissue were not statistically significant.

Exudate was present in the ulcers of all 10 patients at the first visit; in seven cases (70%) it was of serous origin, and in three cases (30%), it was of serosanguineous origin. At the sixth visit, there was no change in the number of patients or the type of exudate; a change was observed only at the 12th visit, when eight (80%) patients had serous exudate and two (20%) had serosanguineous exudate in their ulcers.

The amount of exudate at the first visit was variable in all patients. Three (30%) had a low amount of exudate, six (60%) had a moderate amount of exudate, and one had a high amount (10%). At the sixth visit, there was a change in this pattern: four (40%) patients had a low amount of exudate, and six (60%) had a moderate amount. At the 12th visit, seven (70%) patients had a low amount of exudate, and three (30%) had a moderate amount.

**Figure 2:**
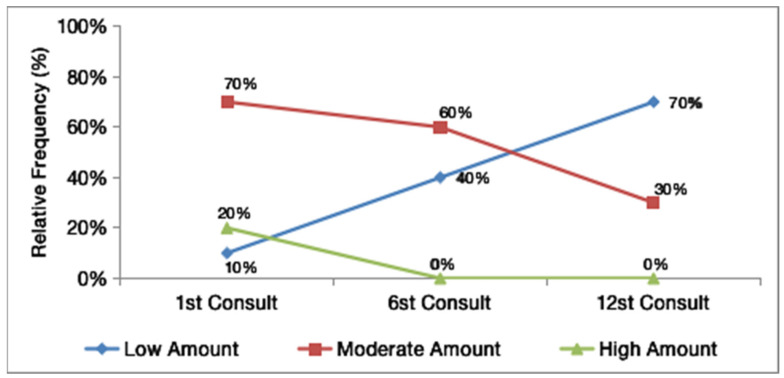
The evolution of the frequencies of each classification of the amount of exudate in the wound bed.

Figure 2 shows the evolution of the frequencies of each classification of the amount of exudate present in the wound bed of the 10 patients over the 12 weeks of treatment. There was mainly a marked growth in the percentage of cases with a low amount of exudate. When the classification of the initial and final amounts of exudate was compared pairwise using the Wilcoxon test, the variation in the amount of exudate across the whole group was statistically significant (*p* = 0.021).

In the qualitative assessment of the impressions of patients of the products, the intervention group reported lower adhesion of the primary coverage to the lesion and consequently less pain.

No participant had severe or local adverse events such as severe pain, eczema, or pruritus during the follow-up period.

## 3. Discussion

At the three evaluations, there was a reduction in the lesion area; while there was a greater reduction in the diabetic wounds, the significance of this difference between the two subgroups could not be confirmed by the tests due to the small size of the subgroups. The reduction in the mean lesion area in patients with diabetic ulcers who used rhEGF was also observed in a systematic review with a meta-analysis that included four studies, all of which showed an increased rate of reduction in the ulcer area [14].

In the present study, the data showed an increase in the granulation tissue and epithelialization and, consequently, an increase in the healing rate. The characteristics of the tissue of the wound bed are important indicators of the healing stage achieved or of complications that may be present [15]. The increase in granulation tissue and epithelialization in the wound bed is positive because it favors tissue repair and, consequently, wound healing [16].

A study conducted with 50 patients divided into two groups showed that the wound healing time among the participants in the rhEGF group was significantly (*p* < 0.0001) lower (±12 days) than that of the control group (±18 days) [17]. Another study conducted in Mexico with 34 patients showed that in the rhEGF group, more ulcers achieved full healing (rhEGF, *n* = 4; placebo, *n* = 0; *p* = 0.033); ulcers in the rhEGF group had a greater decrease in the size of the lesion area (12.5 cm^2^ [rhEGF]) compared to the control group (5.2 cm^2^; *p* = 0.049), and more epithelial islands were present in the wound bed (28% vs. 3%; *p* = 0.025) [18].

At the first visit, all patients had devitalized tissue in more than 25% of the wound area. At the 12th visit, only three patients had devitalized tissue at a proportion equal to or less than 25% of the area. Debridement (i.e., removal of the devitalized tissue) may be considered as an essential factor for enabling deeper contact of the rhEGF with the wound, thus promoting the proliferation and migration of epithelial cells, leading to epidermal regeneration and wound epithelialization [19].

The presence of exudate in the wound bed is characterized as a physiological process that occurs in the inflammatory phase of chronic wounds [20]. The presence of serous exudation in the lesion bed shows the absence of infection and a good prognosis. Thus, improvement in the amount and type of exudate are factors that indicate the evolution of the healing process [21]. An important finding of the present study was that the variation in the amount of exudate across the whole group was statistically significant (*p* = 0.021).

In this study, it was observed that the patients with venous compromise had a mean ankle-arm index (AAI) of 0.94, which is consistent with the literature that suggests that an AAI above 0.9 mmHg is indicative of venous compromise [22,23]. This factor directly affects chronic wound healing, further hindering the healing process.

It is estimated that peripheral arterial disease occurs in 40% of patients with chronic ulcers, delaying healing and consecutively resulting in higher rates of amputation and mortality. Thus, it is necessary to evaluate the AAI [24,25].

According to the American Diabetes Association, for effective control of diabetes mellitus, glycated hemoglobin should be less than 7% [26]. In the present study, the mean glycated hemoglobin level was 7.81, demonstrating inadequate control of the underlying disease, which can cause a delay in the healing process. The worsening of glycemic control during the treatment of chronic wounds significantly decreases the likelihood of wound healing [27].

Clinically, the fact that the oil is incorporated into the gel is beneficial for healing, since the gauze adheres less, causing less pain, and less risk of removing newly formed tissue [28].

No patient presented severe or local adverse events during the follow-up period. This finding is consistent with the literature, which shows that the use of growth factors including EGF in chronic wounds is not associated with local or severe adverse events, confirming the good tolerability and safety of this active ingredient [29].

The growth factor treatment in patients with diabetes may lead to lower overall health costs, accelerating the healing of chronic ulcers in patients with diabetes, reducing the need for hospitalization, and consequently, the costs of treatment [30,31]. The growth factor therapy may be a cost-effective and innovative complement to wound care [30].

### Limitations

The sample size was limited, and a control group was not included; therefore, the results reported here reflect the findings of this study.

## 4. Conclusions

There was a reduction in the lesion area of patients with chronic wounds treated with rhEGF, with a greater reduction in the area of diabetic ulcers than in the area of venous ulcers. There was an increase in the percentage of epithelialization and granulation tissue and a decrease in the percentage of devitalized tissue and the amount of exudate. There were no reports of adverse events.

These results suggest that rhEGF is a promising dressing for the healing of chronic wounds, specifically those with diabetic etiology.

## 5. Materials and Methods

This is a multiple case series with a therapeutic intervention developed at the wound healing outpatient clinic of a federal public university hospital in the state of Rio de Janeiro, Brazil. The research protocol complied with the Declaration of Helsinki and was approved by the Research Ethics Committee of the School of Medicine (Opinion number: 2,189,183), and the participants signed an informed consent form.

The sequentially collected sample consisted of 10 patients with diabetes with chronic wounds including four venous ulcers and six diabetic ulcers. All were treated with a carboximethylcellulose (CMC) hydrogel containing rhEGF (4000 ng/g). rhEGF was incorporated into the CMC gel at room temperature without mechanical stirring. As the active ingredient came as an oily vehicle to avoid its oxidation, when incorporated into the gel, it produced an emulsion, which was stable throughout the study period.

Inclusion criteria: Age older than 18 years regardless of gender; diabetes mellitus, and diabetic ulcer or venous ulcer with an evolution time greater than 12 weeks [32]; lesion size >2 cm² and <100 cm²; granulation tissue and/or epithelialization in the wound bed; and daily availability of a refrigerator at home in which to store the product.

Exclusion criteria: Pregnancy or breastfeeding; suspected malignancy of the lesion; immunosuppressive diseases or current immunosuppressive treatment; infected lesions.

The data collection period lasted from May 2017 to January 2018. The follow-up period was 12 weeks. The dressing was applied weekly at the nursing outpatient clinic and daily at home. The outpatient protocol consisted of clinical evaluation, cleaning with 0.9% saline solution, mechanical debridement when necessary, application of the rhEGF-containing gel, dressing with sterile gauze, hydration of the surrounding skin with a urea-based moisturizing cream, and bandaging with a bandage wrap.

The at-home protocol consisted of cleaning of the wound with 0.9% saline solution, application of the gel with rhEGF, coverage with sterile gauze, hydration of the surrounding skin with a urea-based moisturizing cream, and bandaging of the dressing with a bandage wrap. Patients with venous ulcers used compression therapy when prescribed [22,32].

All patients received the rhEGF-containing gel packed in an airless pump and kept in a refrigerated container. They also received kits containing dressing material, nursing instructions, and a folder explaining how to change the dressing.

Sociodemographic and clinical data were analyzed with descriptive statistics and are presented as graphs and tables and expressed by the mean, standard deviation, coefficient of variation, median, minimum and maximum for numerical data, and by the frequency (*n*) and percentage (%) for the categorical (qualitative) data. The significance of the reduction in the wound area was assessed by the *t* test for means, considering the following null Hypothesis H0: “The mean reduction is equal to 0” (i.e., “There was no significant reduction”). Ordinal data with repeated measurements were compared pairwise with a nonparametric approach using the Wilcoxon test due to the small sample size. The significance level adopted in the analyses was 5% (i.e., the null hypotheses of the tests were rejected if the resulting *p*-value was below 0.05). The analyses were performed using SPSS version 22.0. Microsoft Excel 2011 software, Microsoft, Redmond, WA, USA, was used to build the database and graphs.

## Figures and Tables

**Table 1:** The main statistics of the sociodemographic and clinical variables of the patients at the baseline.

Variable	Category	Absolute Frequency	Relative Frequency
Sex	Female	3	30%
	Male	7	70%
Baseline Diseases	DM *	2	20%
	DM + SAH &	7	70%
	DM + SAH + CVI @	1	10%
Etiology of Injury			
	Diabetic Ulcers	6	60%
	Venous Ulcers	4	40%
Time of the lesion	Up until 1 year	3	30%
	>1 year to 5 years	4	40%
	More than 5 years	3	30%
	**Median**	**Standard Deviation**	**Coefficient of variation**
Age (years)	61.92	9.41	0.15
Time of the lesion (Months)	63.4	74.3	1.17
Body Mass Index (kg/m²)	32.17	6.32	0.20
Glycated Hemoglobin (%)	61.92	2.71	0.04
Ankle-Brachial Index	0.94	0.21	0.22

*—Diabetes Mellitus; &—Systemic Arterial Hypertension; @—Chronic Venous Insufficiency SPSS software, version 22.0, Microsoft, Chicago, United States of America .

**Table 2:** The main statistics for the absolute reduction and relative reduction of the ulcer area.

	Global	Diabetic Ulcers	Venous Ulcers
**Relative reduction (%)**			
Minimum	2.7	3.4	2.7
Median	23.6	23.6	18.9
Maximum	79.8	79.8	48.1
Mean	28.9	33.4	22.1
Standard Deviation	25.9	30.0	20.1
Coefficient of variation	0.90	0.90	0.91
**Absolute Reduction (cm^2^)**			
Minimum	0.2	0.4	0.2
Median	1.3	1.3	1.8
Maximum	38.0	38.0	9.0
Mean	5.6	7.2	3.2
Standard Deviation	11.7	15.1	4.1
Coefficient of variation	2.09	2.10	1.28

SPSS software, version 22.0.

**Table 3:** The frequency distribution of patients according to the percentage of each tissue type present on the first and twelfth weeks of treatment, N = 10.

	Granulation Tissue		Epithelialization Tissue		Devitalized Tissue	
(%)	1st Consult	12st Consult	1st Consult	12st Consult	1st Consult	12st Consult
76–100	30%	10%	0%	0%	0%	0%
51–75	50%	50%	0%	0%	20%	0%
26–50	0%	30%	0%	10%	0%	0%
1–25	20%	10%	0%	30%	50%	40%
0	0%	0%	100%	60%	30%	60%
*p*-valor *	0.463		0.066		0.107	

* According to the Wilcoxon test comparing the two distributions.

## Data Availability

The data presented in this study are available on request from the corresponding author. The data are not publicly available due to maintaining patient privacy.
